# A Compact Measurement Setup for Material Characterization in W-Band Based on Dielectric Waveguides

**DOI:** 10.3390/s22165972

**Published:** 2022-08-10

**Authors:** Kerstin Orend, Christoph Baer, Thomas Musch

**Affiliations:** Institute of Electronic Circuits, Ruhr University Bochum, 44801 Bochum, Germany

**Keywords:** material characterization, dielectric waveguide, 3D printing

## Abstract

In this contribution, we present a measurement system for material characterization in the millimeter-wave range that requires extremely small amounts of sample material. With the help of a dielectric waveguide, it is possible to measure the complete S-parameters with only one port. Fundamentals regarding dielectric waveguides and algorithms are explained, which form the basis of the measurement system. Within the scope of this work, an existing waveguide system was extended and optimized. In addition, two algorithms were implemented to determine permittivity. Finally, measurements were carried out to prove the function of the measurement setup and compared to existing measurement setups.

## 1. Introduction

Non-contact material characterization is an interesting topic for both academic research and industrial applications. In RF systems, dielectric materials are not only mechanical components, but also fulfill functions, such as waveguides or antennas. For the design of such RF components, knowledge of the dielectric material properties in terms of the permittivity ($\varepsilon_{r}$) and loss tangent (tanδ) is therefore very important. Up to now, material properties are often only specified in the data sheet for individual frequency points. Even more interesting is the knowledge of permittivity over an entire frequency band, especially at high frequencies. This allows broadband simulations to be performed for optimizing RF component design. However, also in other research areas, as well as in industry, the knowledge of permittivity is of great interest for manufacturing, quality control, and process monitoring, e.g., pharmacy, biochemistry, the forestry and timber industry, construction, agriculture, trade, and also the food industry [1,2,3,4].

Various methods can be used to measure permittivity in the millimeter-wave range. The method of measurement varies with frequency, e.g., cavity resonators, the transmission line method, and free space setups [5]. The resonator method represents an efficient method for low-loss single-frequency measurements, whereas the free space system is preferred for measurements over a whole frequency band. However, all of these methods are for frequencies below 50 GHz. The material characterization kit (MCK) of “Swissto12” as the gold standard provides a setup in the millimeter-wave (mmW) range above 50 GHz to measure permittivity. The MCK consists of two tapered flared waveguides, with the shaft connected by a corrugated waveguide, forming a low-loss $HE_{11}$ hybrid mode. A variable gap between the two waveguides allows the material samples to be inserted into the system during the measurement. The material under test (MUT) must have a homogeneous thickness of a maximum of 20 mm. There is no strict minimum thickness; ideally, a few millimeters is sufficient for low-loss materials. In addition, it must have lateral dimensions to cover the antenna aperture of typically 40 mm × 40 mm, which corresponds to an area of $16\;{\mathrm{cm}}^{2}$. The waveguide ends are connected to a vector network analyzer (VNA), which measures the S-parameters in the frequency range of the MUT. Permittivity values can be determined from the measured S-parameters of the material under test using iterative algorithms. The setup has an error in permittivity of ±1% [6]. However, the system is very cost-intensive due to the complex manufacturing of the corrugated horn.

In addition to the 2-port measurement systems that evaluate the transmission, there are also 1-port systems that make reflection measurements for high frequencies. For this purpose, a frequency-modulated continuous wave radar system (FMCW) can be used instead of a VNA. Radar systems offer a large dynamic measurement range, while at the same time being compact. By applying calibration techniques from the VNA field, they can also be used to characterize materials with high accuracy. Another MCK is based on such an FMCW radar system. The signals are transmitted and received by means of dielectric lens antennas. An additional ellipsoidal dielectric lens antenna, which is located in the middle of the setup, is used to transform the spherical wavefronts radiated by the antenna to a nearly plane wavefront. The sample is placed at the end of the scaffold. Here, too, a minimum size of approximately 40 mm × 40 mm is required [7]. However, these methods already developed require samples of at least 40 mm × 40 mm in the W-band. This is particularly disadvantageous for very small material samples as they are mandatory for expensive or hazardous materials, such as enzymes or explosives, respectively. To solve the above problems, a measurement concept called the 2-port-1-port system is presented in this paper. It enables transmission measurements of samples with a cross-sectional area as small as $0.25\;{\mathrm{cm}}^{2}$. Beyond that, just one port is necessary. Nevertheless, the complete set of parameters can be measured. The separation of reflection and transmission takes place via lines of different lengths of the DWG. This allows the combination of reflection and transmission methods. The system can be used with both a classic VNA and a more compact FMCW radar system. Furthermore, the complete dielectric waveguide system was printed at low cost with the help of a 3D printer. This manuscript is organized as follows: Section 2 explains the fundamentals of dielectric waveguides and the concept of the 2-port-1-port system and presents algorithms for permittivity calculation. Section 3 shows the simulations of the optimized waveguide system, as well as the coupling with the corresponding results. The calibration of the setup and further processing are shown in Section 4. Section 5 takes a closer look at the tolerances and measurement accuracies. Finally, the measurement setup and results are shown in Section 6. Section 7 concludes the manuscript. In Section 8, the results are discussed.

## 2. Fundamentals

### 2.1. Dielectric Waveguide

**Figure 1:**
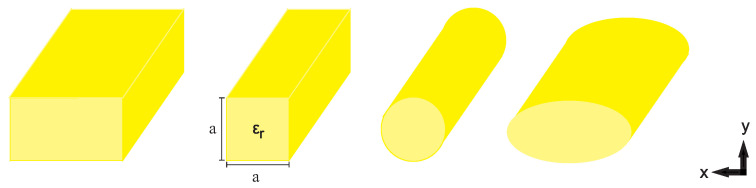
Cross-section geometries of a dielectric waveguide from left to right: rectangular, square, round, and elliptical.

Electromagnetic waves can be guided not only by means of metallic conductive waveguides, but also with non-conducting media. These are called dielectric waveguides (DWG). Four options are available for selecting the cross-sectional geometry of the dielectric waveguide as shown in Figure 1: rectangular, square, round and elliptical.

However, the latter has proven advantageous, because here, the polarization of the guided wave is maintained.

**Figure 2:**
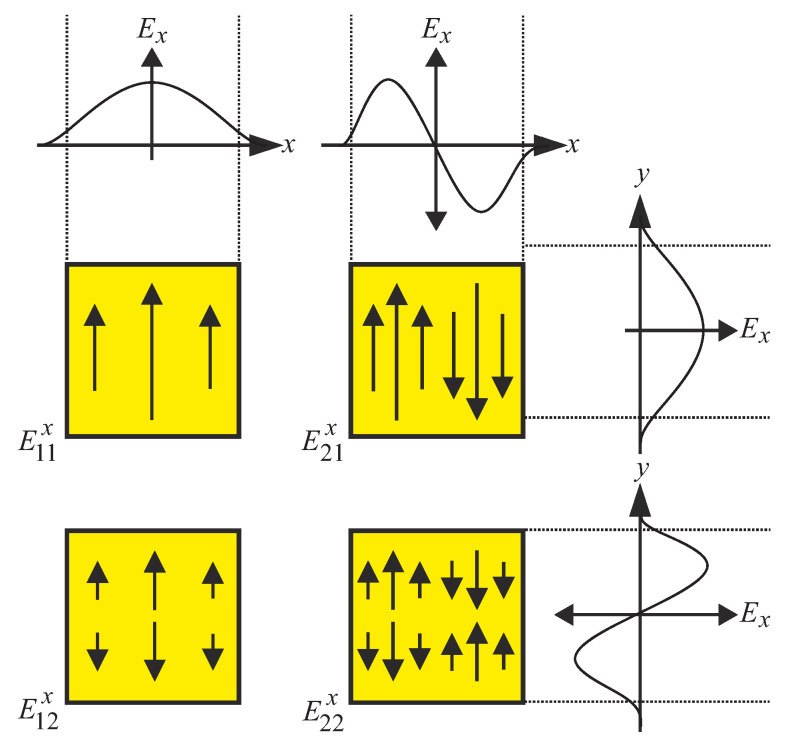
Schematic illustration of the first modes of a quadratic DWG in x-polarization $E_{mn}^{x}$.

If a polarimetric approach is followed, a square DWG cross-section is preferred. There are a certain number of propagation modes for DWGs. Most of these modes have electric and magnetic field components in all three spatial directions and are called hybrid modes. The modes of the electric field are denoted as $E_{mn}^{x}$ or $E_{mn}^{y}$. The superscript indicates the direction of the E-field, while the index indicates the number of field maxima in both directions. Figure 2 shows the field distribution of the first modes of a quadratic DWG in x-polarization $E_{mn}^{x}$. The fundamental modes of a square DWG show perfect orthogonality in $E_{11}^{y}$ and $E_{11}^{x}$. Comparing the field distributions in the xy-plane with those of an $H_{10}$-mode in the waveguide, we see a great similarity of the spatial field strength distribution.

**Figure 3:**
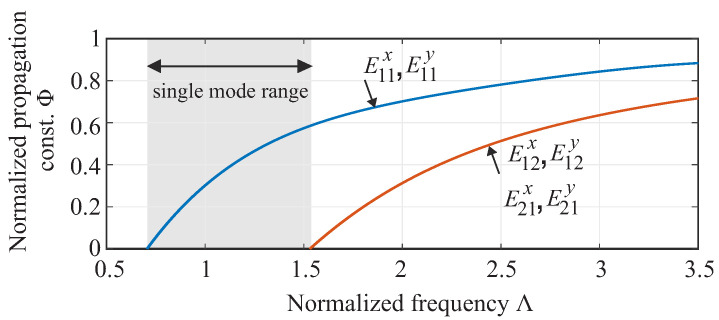
Plot of propagation constant versus normalized frequency for the first two, square DWG modes and implied mono-mode area.

When choosing the appropriate mode, it is important that it is easy to excite and transmits the wave as loss-free as possible. Furthermore, it is important that the propagation is single mode over a wide frequency range. Figure 3 shows the mode diagram over the normalized frequency of the first two excitable modes, on a square DWG. The normalized frequency Λ is given by the following equation:

(1) $$\Lambda =\frac{2a}{\lambda }\sqrt{\varepsilon_{r,\mathrm{DWG}}-\varepsilon_{r,\mathrm{Air}}}$$

where a is the width of the DWG, λ is the wavelength, $\varepsilon_{r,\mathrm{DWG}}$ is the permittivity of the waveguide, and $\varepsilon_{r,\mathrm{air}}$ is the permittivity of the air. Consequently, the permittivity, as well as the cross-sectional edge length a of the DWG determine the single-mode frequency range. Moreover, it can be seen from the diagram that the square DWG shape provides a wide single-mode range, which is useful for the development of broadband measurement systems. The following condition must be fulfilled for the single-mode range:

(2) 0.74<Λ<1.51

Hence, a 2 mm × 2 mm DWG has a single mode range of 46–92 GHz for the fundamental mode $E_{11}^{x}$, respectively $E_{11}^{y}$. Below this frequency, the propagation of electromagnetic waves is no longer possible, and they radiate into free space [8,9]. Dielectric waveguides do not have a cutoff frequency, as is known from waveguides. Nevertheless, a characteristic frequency can be determined—the so-called divergence frequency. The divergence frequency of the propagation mode is indicated by the zero crossings of the propagation constant and is for the $E_{11}$ mode at Λ=0.74.

**Figure 4:**
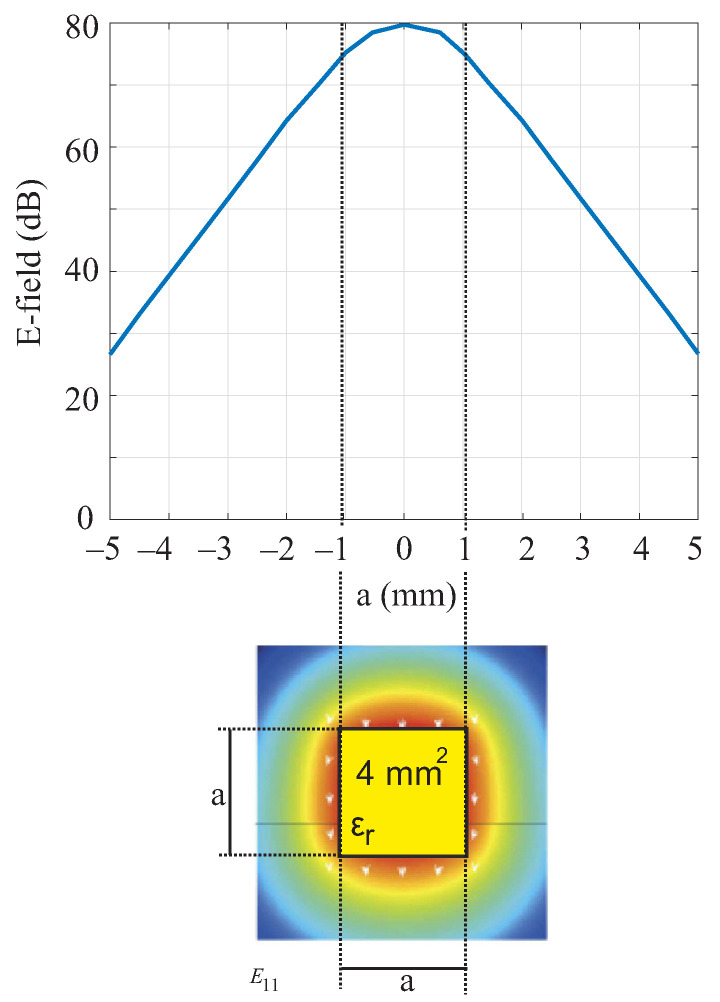
Simulated E-field distribution of the fundamental mode $E_{11}^{x}$ of a quadratic DWG.

Due to the small cross-section of the dielectric waveguide, reliable measurements can be obtained even with small amounts of the material under investigation. This is an advantage for explosive and very expensive materials. The reason is that, despite the small cross-section, most of the field is propagated in the DWG. Figure 4 shows the simulated E−field of the fundamental mode $E_{11}^{x}$. Towards the edges, the field strength drops by 8 dB. At a distance of 2 mm from the DWG, the field strength drops by 29 dB.

For the construction of a flexible dielectric waveguide, thermoplastics are particularly well suited. Mainly, high-density polyethylene (HDPE) and high-impact polystyrene (HIPS) are used. These materials consist of non-polar molecules and, thus, have a low dielectric loss factor [10]. Furthermore, these materials are characterized by the following properties:

- Low weight;
- Flexibility due to their bendability;
- Easy fabrication, as well as length adaptation;
- Mechanically stable;
- Low material and manufacturing costs.

Due to their properties, DWGs represent an efficient alternative to conventional metallic waveguides and quasi-optical shaft guides.

### 2.2. Concept

**Figure 5:**
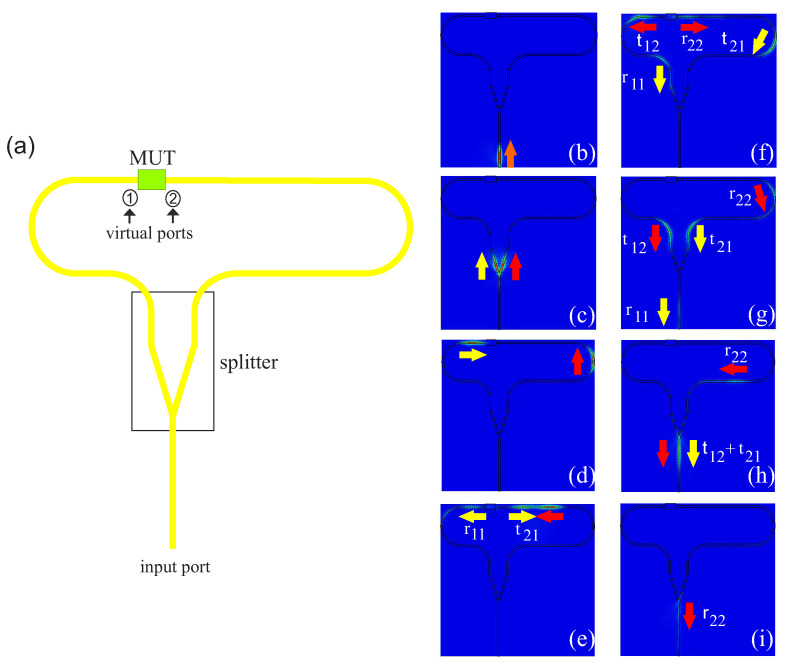
(**a**) shows the schematic structure. (**b**–**i**) illustrate the signal step by step using field recordings at different times.

In order to reduce the dimensions and complexity of the usual measurement setups, a measuring concept called the 2-port-1-port system was presented in [11]. This setup requires only one measuring port. This means that reflection and transmission can no longer be separated from each other via the ports. However, if a reflectometer with sufficient bandwidth is available, the signals can be separated from each other via the time domain. This can be realized by the setup in Figure 5a. The setup consists of a dielectric waveguide, which splits into two paths of different lengths. The sample to be measured is placed between the two open ends. On the right-hand side of Figure 5, the signal curve is illustrated step by step using field images. With a reflectometer, a signal is fed into the waveguide; see Figure 5b. Most of the field propagates in the dielectric waveguide. The orange arrow indicates the direction in which the wave propagates. Figure 5c shows that the wave is separated at the splitter. The left signal part is marked with a yellow arrow and the right with a red one. Due to the different lengths of the paths, the signal takes different lengths of time to reach the MUT. As shown in Figure 5d, the signal marked with a yellow arrow reaches the virtual port 1 first. The signal arriving at the MUT is partly reflected and partly transmitted in the next step. The ratio in which parts are reflected and transmitted depends largely on the material properties of the MUT. The reflected part of the yellow marked signal at the virtual port 2 is called $r_{11}$, and the transmitted part is called $t_{21}$, as illustrated in Figure 5e.

The time-delayed signal arriving at virtual port 2, which is marked by a red arrow, is also partially reflected and transmitted. The reflected signal is labeled as $r_{22}$ and the transmitted signal as $t_{21}$; see Figure 5f. The reflected signal $r_{11}$ reaches the input/output port first, since it has to propagate the shortest distance, as illustrated in Figure 5g. The transmitted signal components reach the input/output port in time, so that the signal components perfectly overlap. This doubles the amplitude; see Figure 5h. The last Figure 5i shows that the reflected signal from virtual port 2, $r_{22}$, arrives last due to the longest distance. As a result, the time signal is composed as follows:

(3) $$\tau \left(t\right)=s(A_{\mathrm{fs}}p\left(t\right)+\underset{r_{11}}{\underset{︸}{A_{11}p\left(t-t_{1}\right)}}+2\underset{t_{12}+t_{21}}{\underset{︸}{A_{12}p\left(t-t_{2}\right)}}+\underset{r_{22}}{\underset{︸}{A_{22}p\left(t-t_{3}\right)}}$$

It is a composition of the reflection and transmission signals, where *s* is an amplitude factor, $A_{ij}$, i,j = 1, 2 the amplitude, $A_{\mathrm{fs}}$ the feed-side attenuation of the coupling, p(t) the pulse shape, and $t_{i}$, *i* = 1, 2, 3 the different travel times of the pulses. The propagation times depend directly on the line length *l*, the thickness *d* of the MUT, and the propagation velocity in the respective medium. The transit times $t_{i}$ of the different sections are defined as follows:

(4) $$t_{0}=2\cdot \frac{l_{01}}{c_{1}}$$

(5) $$t_{1}=t_{0}+2\cdot \frac{l_{1}}{c_{1}}$$

(6) $$t_{2}=t_{0}+\cdot \frac{l_{1}+l_{2}}{c_{1}}+\frac{d}{c_{2}}$$

(7) $$t_{3}=t_{0}+2\cdot \frac{l_{2}}{c_{1}}$$

**Figure 6:**
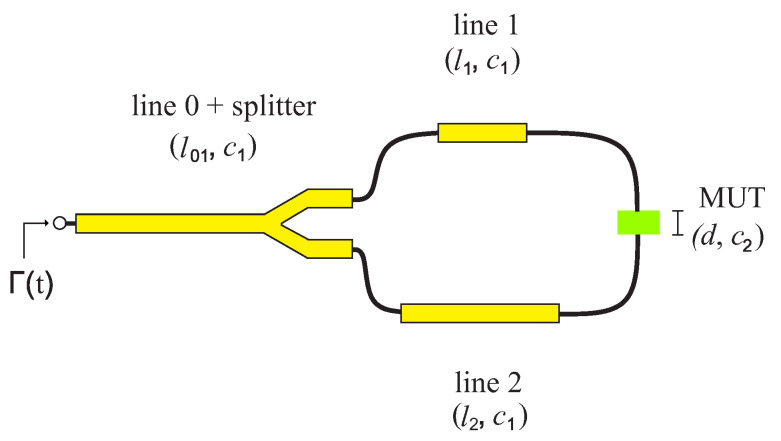
Schematical drawing.

Figure 6 shows a schematic drawing of the 2-port-1-port system, illustrating the propagation speeds in the respective sections including the lengths.

**Figure 7:**
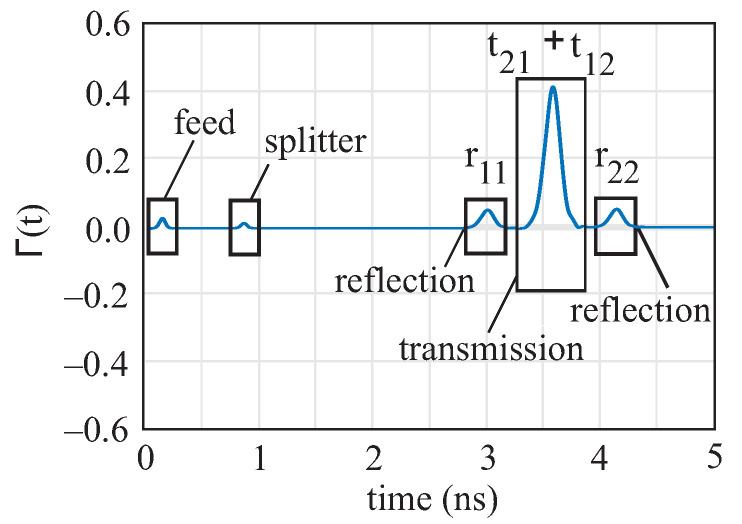
Measured time domain signal τ of a Teflon sample.

An example of the Hanning-windowed time domain signal of the waveguide system is shown in Figure 7. At the time instants 2.9 ns and 4.2 ns, the signal pulses of the two reflections can be seen. The highest peak at 3.6 ns, located in the middle of the two reflection signals, represents the superimposed transmission signal. At the earlier times, 0.1 ns and 0.9 ns, smaller pulses can be observed, which appear due to the feeding of the signal and the splitter.

To prevent the transmission peak $t_{21}/t_{12}$ from overlapping with the reflection peaks $r_{22}/r_{22}$, only samples of thickness d and permittivity $\varepsilon_{r,\mathrm{DWG}}$ that satisfy the following condition can be measured:

(8) $$\left(\frac{l_{2}-l_{1}}{\frac{c}{\sqrt{\varepsilon_{r,\mathrm{DWG}}}}}\right)-t_{p}>\frac{\Delta d}{\frac{c}{\sqrt{\varepsilon_{r,\mathrm{MUT}}}}}$$

$l_{1,2}$ stands for the length of subsections of the DWG, $t_{p}$ for the pulse width, *d* for the thickness of the MUT, c for the speed of light, $\varepsilon_{r,\mathrm{DWG}}$ for the permittivity of the DWG, and $\varepsilon_{r,\mathrm{MUT}}$ for the permittivity of the MUT.

### 2.3. S-Parameters’ Extraction

**Figure 8:**
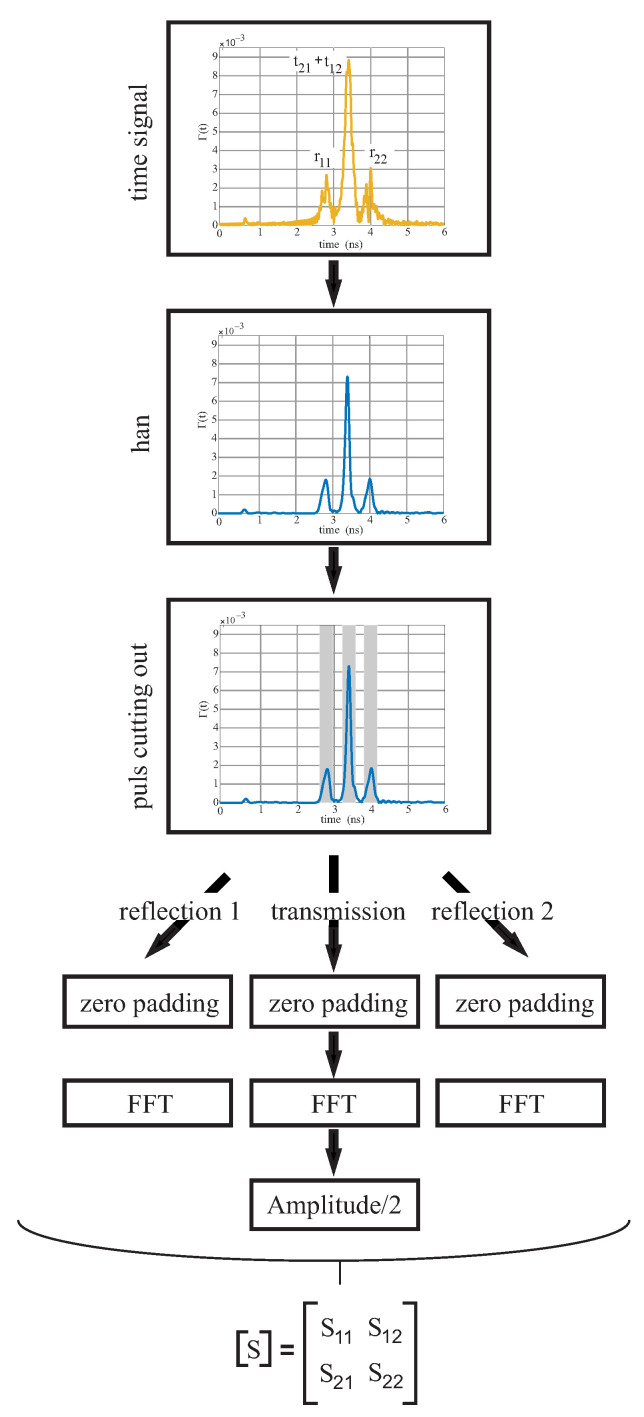
Extraction of the S-parameters.

To determine the permittivity of the sample using the NRW/Baker Jarvis algorithms, the complete S-parameter set is needed. Since the setup is a one-port system, the reflection and transmission factors must be extracted from the time domain signal. Figure 8 shows the procedure for extracting. In addition, the time gating areas are indicated by a gray shading.

First, the time signal is multiplied by the Hanning function. This simplifies the determination of the high points (HPs) and reduces overlapping side slots. Next, the pulses are cut symmetrically around the HP with Δ N and expanded to $2^{15}$ values using zero padding. Zero padding before an FFT is a computationally efficient method for interpolating a large number of points. A complex FFT transforms the data from the time domain to the frequency domain. Since the transmitted pulse is the superposition of $S_{21}$ and $S_{12}$, the amplitude must be divided to determine both values.

### 2.4. Algorithm

**Figure 9:**
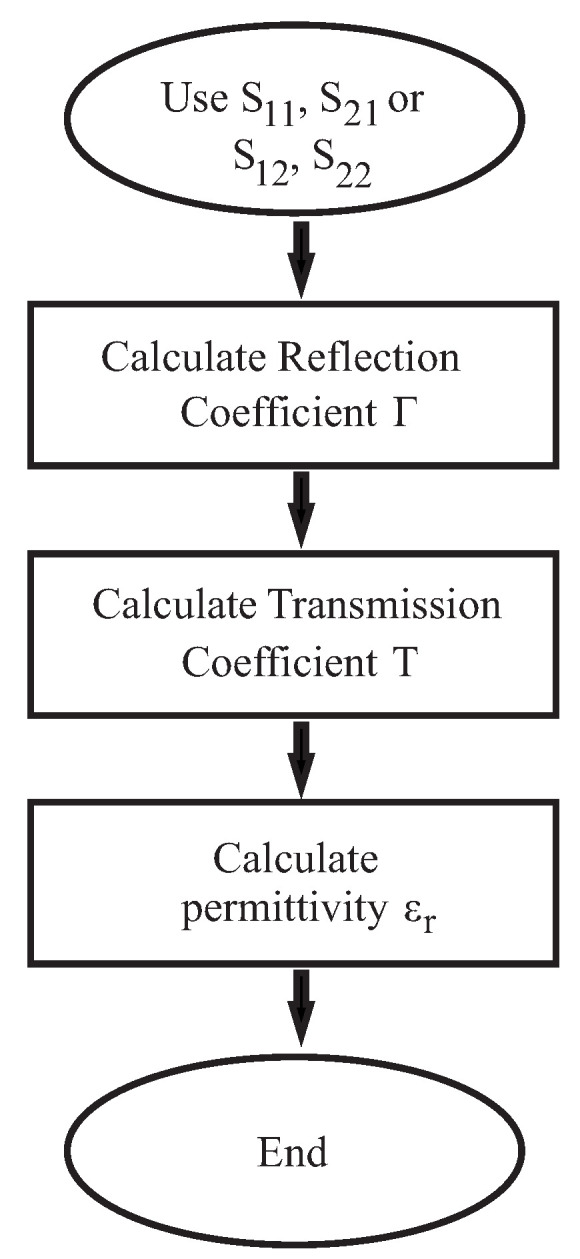
Flow chart of the NRW algorithm.

A number of methods exist for measuring permittivity and permeability. One of the best-known methods is the Nicolson–Ross–Weir algorithm (NRW) [12,13]. Using the Nicolson–Ross–Weir algorithm, the relative permittivity and relative permeability of a material can be calculated by measuring the reflection and transmission of an electromagnetic wave. In principle, it is possible to obtain unambiguous results with a priori information about the sample length d. Figure 9 shows the process of the NRW method.

First, the reflection coefficient is calculated, where |Γ| < 1 is required to obtain the correct result.

(9) $$\Gamma =\chi \pm \sqrt{\chi^{2}-1}$$

χ can be calculated via Equation (3). The S-parameters are extracted from the VNA/radar measurement.

(10) $$\chi =\frac{S_{11}^{2}-S_{21}^{2}+1}{2S_{11}}$$

The transmission coefficient is given as:

(11) $$T=\frac{S_{11}+S_{21}-\Gamma }{1-\left(S_{11}+S_{21}\right)\Gamma }$$

Provided that the permeability is equal to 1 + i0, the permittivity $\varepsilon_{r}$ can be calculated with the help of the free space wavelength $\lambda_{0}$ and the cutoff wavelength $\lambda_{c}$.

(12) $$\varepsilon_{r}=\lambda_{0}^{2}\left(\frac{1}{\lambda_{c}^{2}}-{\left[\frac{1}{2\pi d}\ln\left(\frac{1}{T}\right)\right]}^{2}\right)$$

The cutoff wavelength $\lambda_{c}$ corresponds to the wavelength of the divergence frequency. Equation (5) has an infinite number of solutions, since the imaginary part of the term ln(1/T) corresponds to j(ϕ + 2*n*). *N* can take values of 0, ±1, ±2 …For thin samples, where *d* is less than or equal to half lambda, *n* = 0; for all others, *n* must be calculated. The unwrapping method can be used to determine *n* [14].

**Figure 10:**
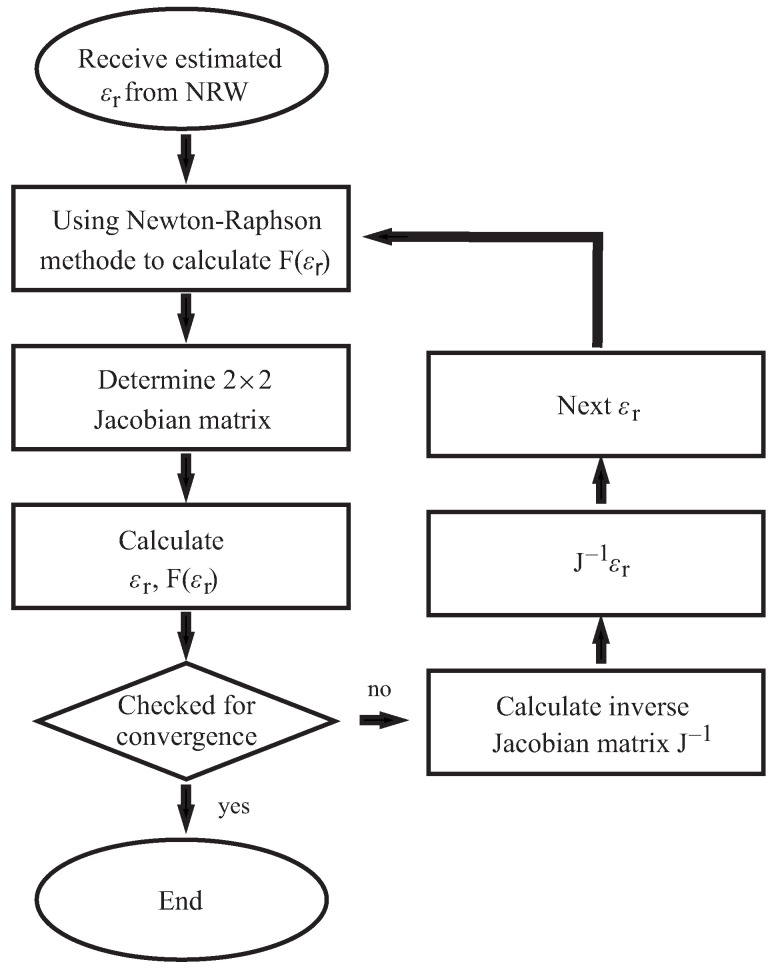
Flow chart of the NIST algorithm.

Another well-known algorithm was developed by Baker–Jarvis in 1990 and is called the NIST algorithm in the literature [15]. In contrast to the NRW algorithm, it is iterative. Figure 10 shows the process for the NIST method.

The method requires an initial value. The NRW method is often used to determine the initial value. With the NIST algorithm, the reflection coefficient is also calculated first.

(13) $$\Gamma =i\left(\frac{2\pi }{\lambda_{0}}\right)\sqrt{1-{\left(\frac{\lambda_{0}}{\lambda_{c}}\right)}^{2}}$$

The transmission coefficient is calculated as follows:

(14) $$T=e^{-id\left(\sqrt{\varepsilon_{r}\varepsilon_{0}\mu_{0}\omega^{2}-{\left(\frac{2\pi }{\lambda_{c}}\right)}^{2}}\right)}$$

By solving Equation (7) using Newton’s method, the permittivity can be determined. The solution is found when the equation converges to 0.

(15) $$F\left(\varepsilon_{r}\right)=\frac{S_{12}+S_{21}}{2\left(1-T^{2}\Gamma^{2}\right)}-T\left(1-\Gamma^{2}\right)$$

This equation assumes a permeability of the material under investigation of 1 + i0.

## 3. Simulation-Based Design

Several 3D EM simulations were performed in CST Microwave Studio 2021 in order to optimize the design of the 2-port-1-port system.

### 3.1. Support Structures

**Figure 11:**
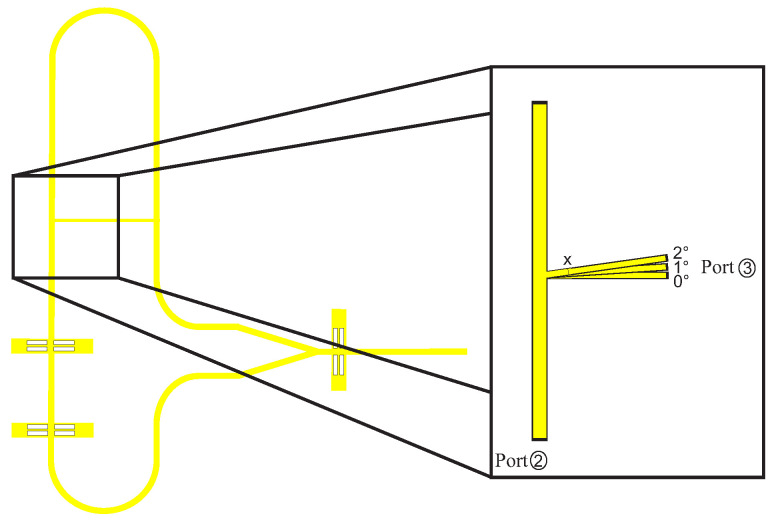
Enlarged illustration of the transition from the dielectric waveguide to the support structure with three different angles of inclination.

Support structures were integrated to stabilize the waveguide structure. Stabilization is important to avoid distortion of the DWG. At very high frequencies, even moving the dielectric waveguide can cause changes that affect the results of the measurement with the VNA. At the same time, mechanical stabilization increases reproducibility. The support structures are also made of HIPS, which allows them to be printed in time with the 2-port-1-port system. Figure 11 illustrates the geometry and positioning of these support structures.

**Figure 12:**
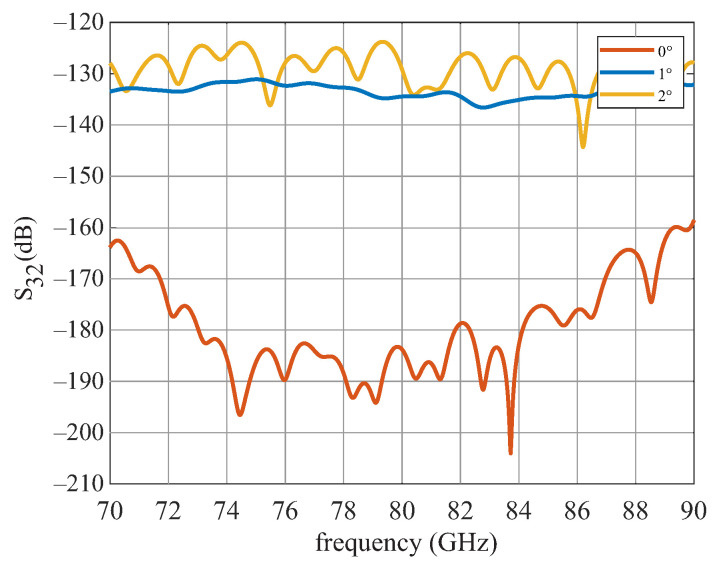
Simulated transmission $S_{32}$ for three angles.

**Figure 13:**
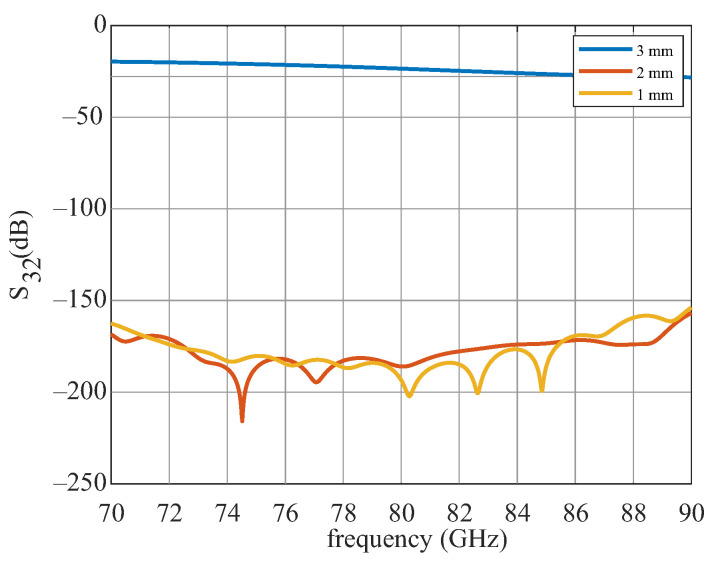
Simulation results of the transmission $S_{32}$ for three support structures.

When positioning and geometrizing the support structures, it is important that they do not influence the actual signal path on the DWG. This means that as little field as possible should propagate in the support structures. Unwanted field components can lead to a decrease in amplitude, as well as to the fact that the reflection signals and transmission signals can no longer be separated from each other. In the first step, the angle of the support structure to the DWG was investigated. Figure 11 shows the simplified simulation model. A wave is fed into the DWG at port 2, and it is seen how much of the signal arrives at port 3. This was carried out for angles of 0°, 1°, and 2°. Figure 12 shows the simulation results for the different angles. For 0°, the coupling is better then 160 dB. At an angle of 1°, the decoupling is already 40 dB worse and lies at −135 dB and for 2°, at −125 dB. This means that the support structure should be exactly perpendicular to the DWG in order to achieve the best result. The height was adjusted to the height of the DWG, so that the drawing forces are evenly distributed and nothing warps. The width *x* of the support structure was also examined. The simulation results are shown in Figure 13.

Here, decoupling was recorded for 1 mm, 2 mm, and 3 mm wide support structures, all perpendicular to the DWG. It can be clearly seen that as soon as the support structure is wider than the DWG, the decoupling decreases significantly by 120 dB. A support structure perpendicular to the DWG with an area of 1 mm × 2 mm was found to be the most suitable, since it has both the necessary stability, as well as low crosstalk.

### 3.2. Mounting

**Figure 14:**
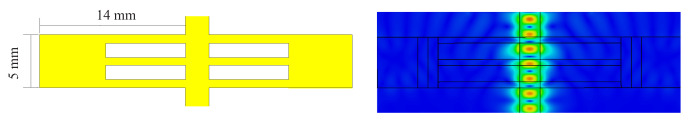
On the right, the simulation model of the mounting structure; on the left, the corresponding field image.

In addition to the support structures, holders have been designed for mounting on optical tables. These are also made of HIPS, which has the advantage that they can be printed in one step together with the 2-port-1-port system. Due to the fact that the mountings are also located on the DWG, it is also important here that as few fields as possible propagate in it. At the same time, it must be stable enough to withstand the mechanical stresses. Figure 14 shows the simulation model and the corresponding field images.

The holder consists of three narrow strips on each side. The number of strips represents a good compromise between material cost, manufacturing cost, stability, and performance. The strips are attached perpendicular to the DWG and have a width of 1 mm. The three strips end in a square piece of hips. This serves to fix the holder with a screw. As can be seen, the majority of the wave propagates in the DWG. The holders are placed in three locations. The first is at the feeder to ensure a tight fit at the transition from the coupling structure to the DWG. The other two are used to position the DWG precisely and stably on the MUT.

### 3.3. Extension

**Figure 15:**
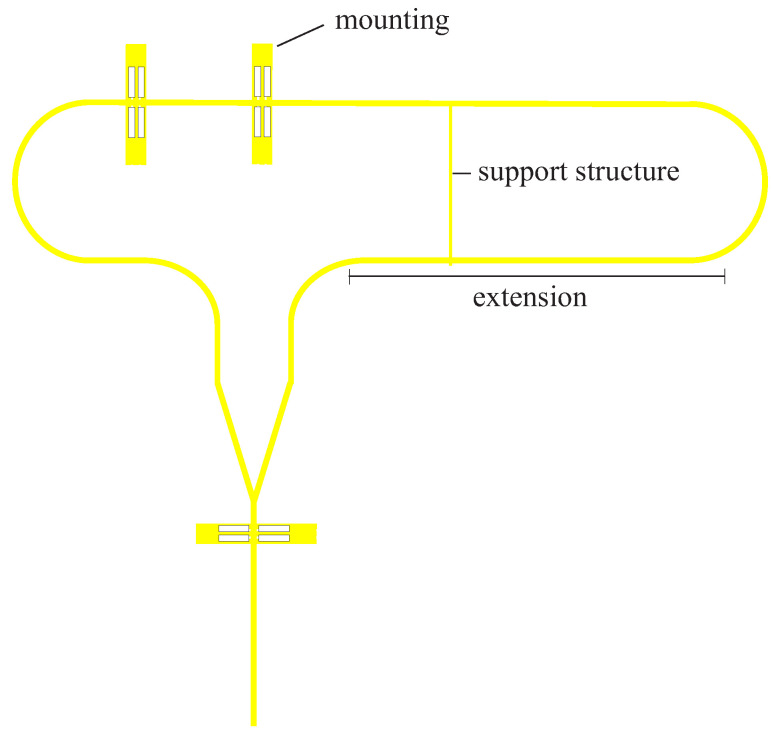
Simulation model of 2-port-1port system with extension.

It is important that the two reflection pulses can be clearly separated from the transmission pulse. Especially for very thick samples, the transmission pulse shifts to the right in the direction of the second reflection pulse. This effect is intensified for samples with high permittivity. At the same time, the ringing increases with increasing permittivity. If the pulses overlap, the S-parameters of the specimen could no longer be extracted, and it would therefore not be possible to calculate the permittivity. To prevent this, the 2-port 1-port system was extended by a 9 cm section. This results in the second reflection pulse being shifted by approximately 1 ns. The pulses are thus further apart in time. This makes it possible to measure both thicker samples and samples with higher permittivity. The simulation model of the 2-port-1-port system with extension is shown in Figure 15.

**Figure 16:**
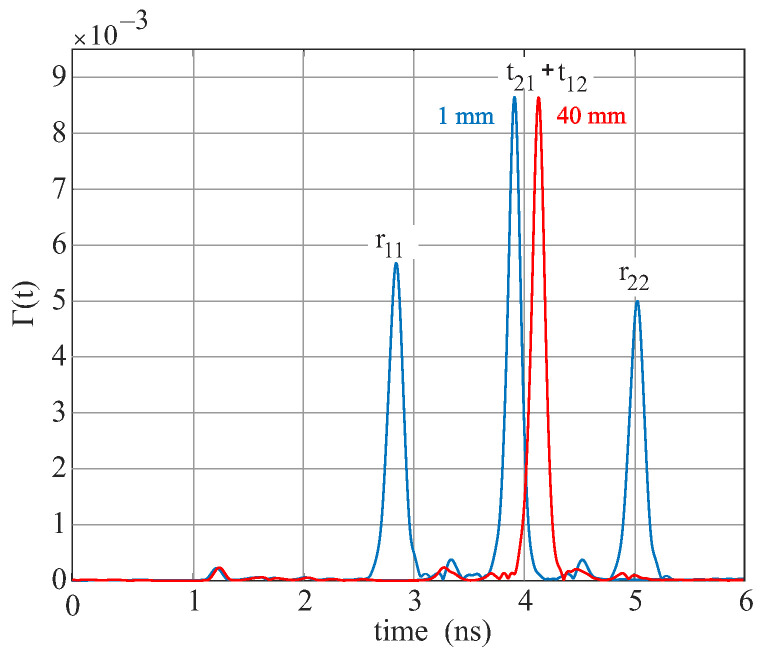
Hanning-filtered time signal for a MUT with 1 mm thickness, as well as with 40 mm.

Figure 16 shows an example of a time signal for a plastic sample with a dielectric constant of 2.5 and a thickness of 1 mm and 40 mm. The two reflection pulses are always at the same position. The first pulse is at 2.9 ns and the second at 4 ns. The transmission pulse, on the other hand, moves to the left as the thickness of the MUT increases. The runtime $t_{2}$ of the transmission is described by the following formula:

(16) $$t_{2}=\frac{s}{\frac{c}{\sqrt{\varepsilon_{r,\mathrm{DWG}}}}}+\frac{\Delta d}{\frac{c}{\sqrt{\varepsilon_{r,\mathrm{MUT}}}}}$$

*s* stands for the length of the DWG, *d* for the thickness of the MUT, *c* for the speed of light, $\varepsilon_{r,\mathrm{DWG}}$ for the permittivity of the DWG, and $\varepsilon_{r,\mathrm{MUT}}$ for the permittivity of the MUT. Due to the mechanical stress on the waveguide during spreading, samples up to max. *d* = 20 mm are possible.

With the extension, samples up to 20 mm with a permittivity between 1 and 80 can be measured.

### 3.4. Transition

**Figure 17:**
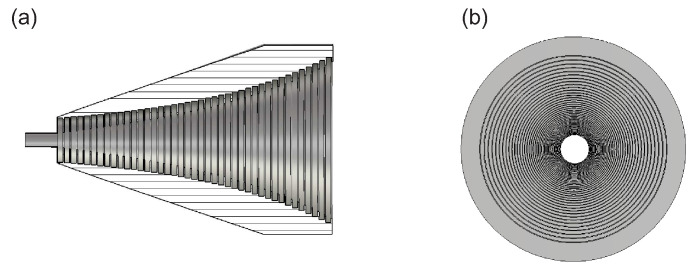
Model of the corrugated horn. In side view (**a**) and in frontal view (**b**).

Due to the similarity between the $H_{10}$ wave in the rectangular waveguide and the $E_{11}^{x}$/$E_{11}^{y}$ wave in the DWG, it is convenient to connect the dielectric waveguide to the network analyzer using waveguides. To make the transition between the two systems as reflection-free as possible, a coupling structure is necessary. This coupling structure should fulfill a number of conditions. At the transition, the wave impedance of the incoming wave should be as close as possible to the wave impedance of the wave to be excited. Furthermore, the transition should be gradual to achieve a smooth adaptation of the wave impedance. This can be advantageously implemented by a horn transition. A schematic sketch of a corrugated horn antenna is shown in Figure 17. There are many horn antennas in the microwave range, but such corrugated horns are characterized by their good matching in a wide frequency band and their low cross-polarization. Furthermore, the field distribution in the horn is similar to that of the DWG.

**Figure 18:**
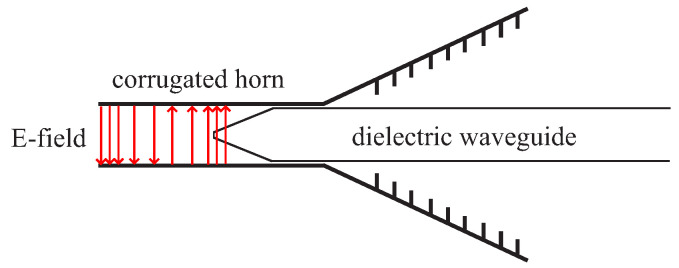
Schematic drawing of the transition from waveguide to dielectric waveguide.

The function of the transition is to transfer the field of the $H_{10}$ wave, which is limited to the waveguide, to the field of the $TE_{11}$ wave, which is partly guided into outer space. A continuous transition is achieved by the horn-shaped opening of the waveguide and by precise insertion of the dielectric line into the waveguide. To reduce the reflections at the interface of dielectric and air, the face of the dielectric waveguide is sharpened. Figure 18 shows a schematic drawing. This modification of the end shape is called tapering. This results in a continuous transition.

**Figure 19:**
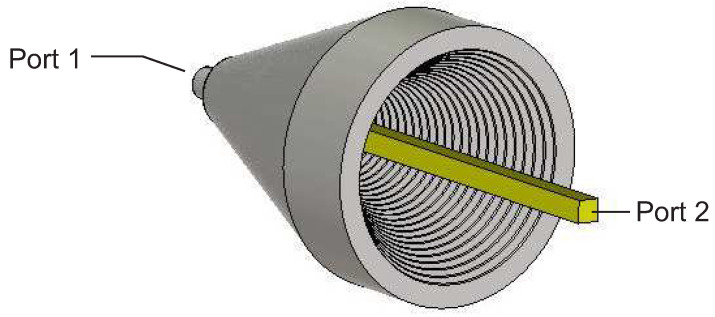
Transition simulation model.

**Figure 20:**
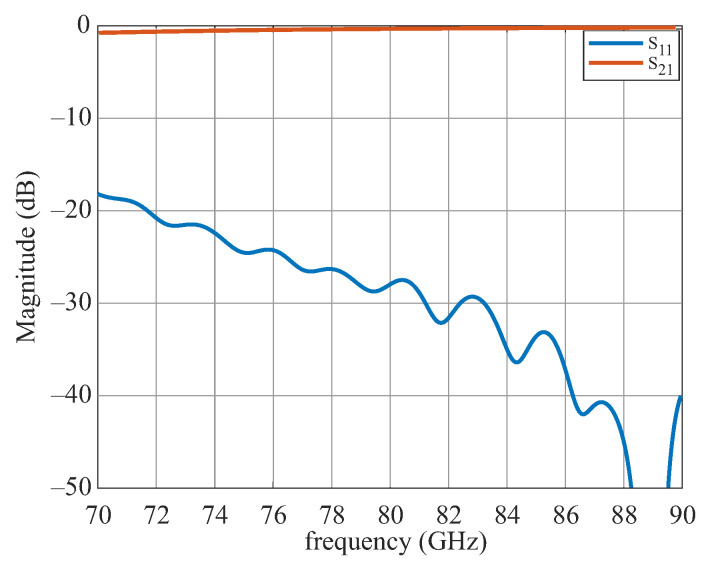
Simulation results of the corrugated horn.

In this work, a corrugated horn coupling for a dielectric waveguide with a rectangular cross-section was designed using CST Microwave Studio. To keep the resulting total attenuation of the transition as low as possible, the optimum horn aperture angle, aperture diameter, and stage design were optimized. Figure 19 shows the simulation model. The coupling structure has a size of 3 cm × 2.5 cm × 4 cm. The corresponding simulation results of the scattering parameters are shown in Figure 20. The forward reflection $S_{11}$ exhibits a good return loss of better than 18 dB for the whole frequency range. The attenuation $S_{21}$ is about 1 dB.

## 4. Processing and Calibration

**Figure 21:**
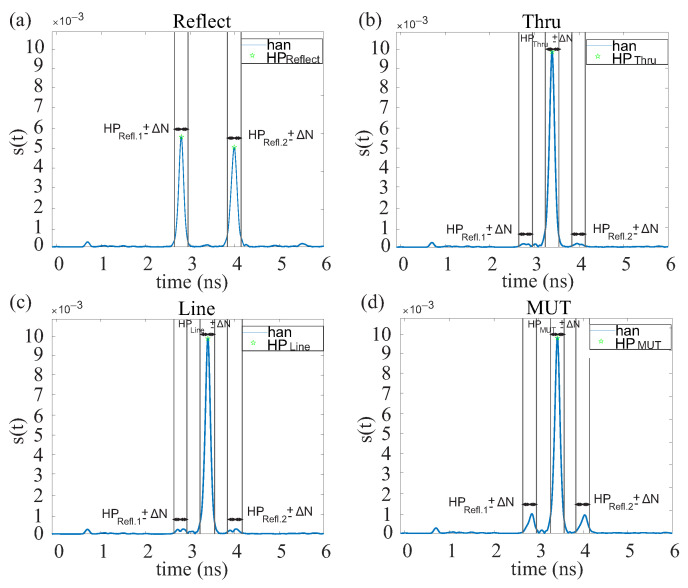
Time domain signals for a reflect standard (**a**), thru standard (**b**), line standard (**c**) and a MUT (**d**).

One of the most important tasks is the extraction of the scattering parameters of the MUT. With their help, conclusions can be drawn about the material properties. In order to extract the S-parameters, calibration must first be performed. Since the measurement signals are guided to the MUT and back again via dielectric waveguides with non-ideal properties, systematic errors occur during the analysis in addition to random errors. The systematic errors can be compensated by calibration measurements. Many methods and strategies exist, which differ considerably in the scope of the error model and, thus, in effort and performance. In the present case, the two-port calibration method through-reflect-line (TRL) was applied. It is suitable due to its wide bandwidth and ease of integration into the structure. To realize the reflect standard, a metal plate is placed between the ports. The through standard is realized by pushing the two ports together. The line standard requires a 1 mm-long piece of dielectric waveguide. With this calibration method, the reference plane is located directly at the virtual ports. Since the reflected pulses are always at the same position, the position is saved and applied to the line and through standard, whereas with the line and through standard, the position of the high point is determined anew since the pulse is slightly shifted. The corresponding time signal is shown in Figure 21.

**Figure 22:**
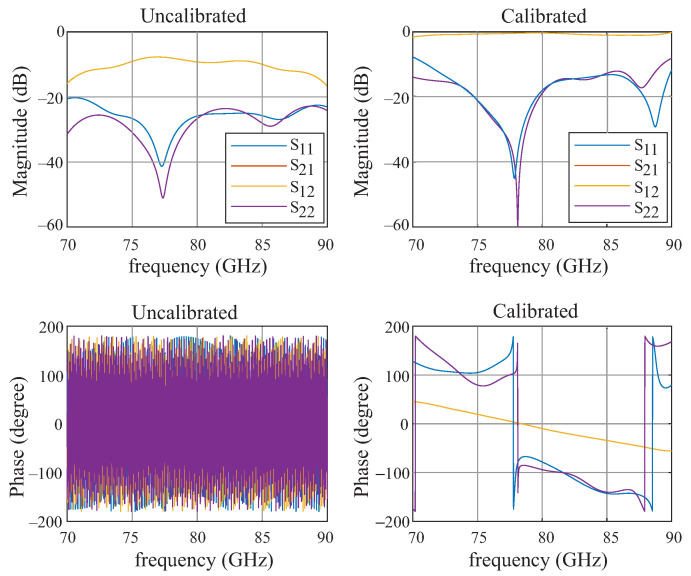
Frequency behavior of the S-parameter. The plot shows the calibrated (**right side**), as well as the uncalibrated (**left side**) result of a lossless 2 mm thick sample with a permittivity of 4.

Since the setup has only one physical port, the S-parameters of the individual standards must be extracted before calibration can be performed. The detailed procedure is described in Section 2.3. The error model extracted from the three aforementioned TRL measurements s applied to the measurement of the MUT. Figure 22 shows both the calibrated, as well as the uncalibrated simulation data of a lossless 2 mm thick sample with a permittivity of 4. The permittivity is calculated from the calibrated S-parameters of the MUT.

## 5. Tolerance and Accuracy

Tolerance refers to the total permissible deviation of an object. It is usually expressed as a (+/−) value related to a given nominal value. In 3D printing, objects can deform as a result of changes in temperature or humidity. These changes cause the material to expand or contract. The deviation tolerance is here about 0.1 mm, but has no influence on the accuracy of measurement.

**Figure 23:**
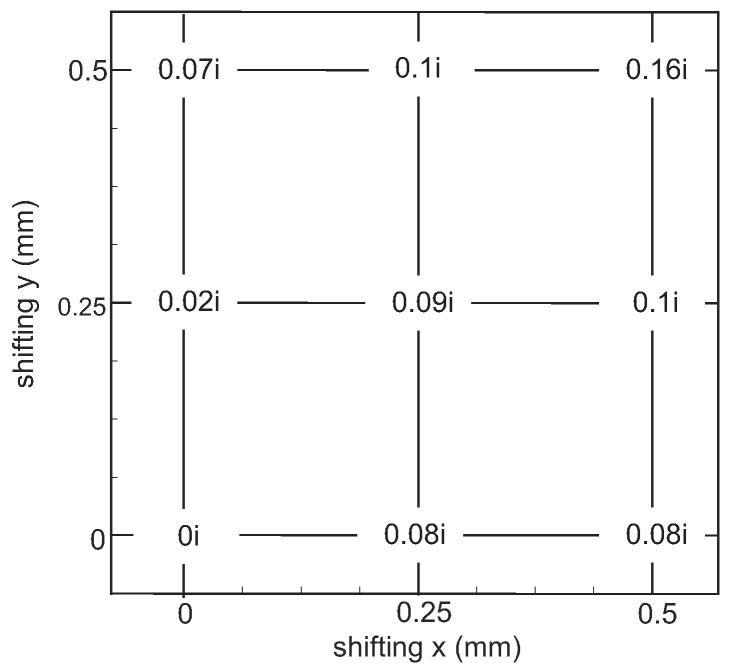
Influence of shifts in the xy plane on the losses.

The measurement accuracy of the losses depends on how accurately the measurement ports are placed relative to each other. A shift causes field components at the transition (DWG–sample–DWG) to be radiated into free space instead of propagating further in the DWG. This reduces the field strength in the DWG, which increases the losses. A shift can occur in the x-, as well as in the y-direction or in combinations of both. This can occur because the DWG is a flexible structure. However, also the holding structures made of the same material are not completely rigid. Figure 23 shows the influence of a displacement on the losses of a 2 mm-thick material with a permittivity of 6. The data are given as the difference from the ideal position. Depending on the material measured, the calculated losses due to the displacement turn out differently.

## 6. Measurements

### 6.1. Measurement Setup

**Figure 24:**
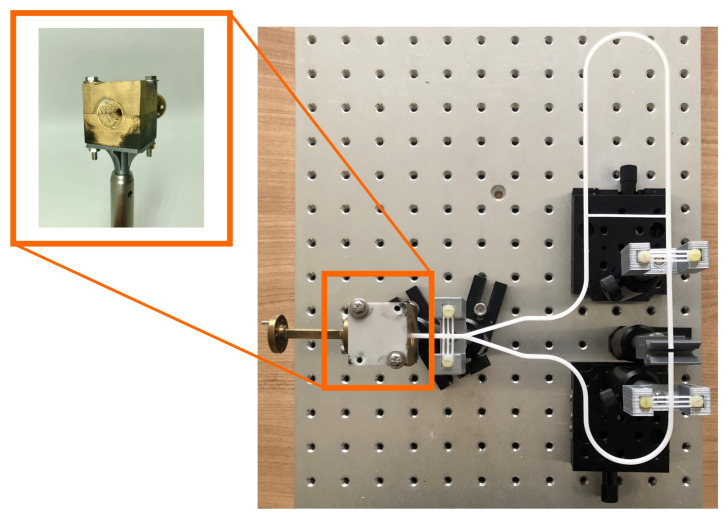
Measurement setup.

The measuring system shown in Figure 24 is set up on an optical measuring table. The rod holders, which are mounted on the optical plate, are variable in height. The coupling structure, the waveguide, as well as the sample holder are screwed to the rod holders to give stability to the system. Two of the rod holders are mounted on sliding tables, which can be adjusted very precisely via a rotary wheel to allow very precise adjustment of the waveguide ends on the MUT. The probe is placed between the two open DWG ends. The maximum specimen thickness depends largely on how much the dielectric waveguides can be spread. At maximum expansion, a MUT with a thickness of approximately 20 mm can be measured.

**Figure 25:**
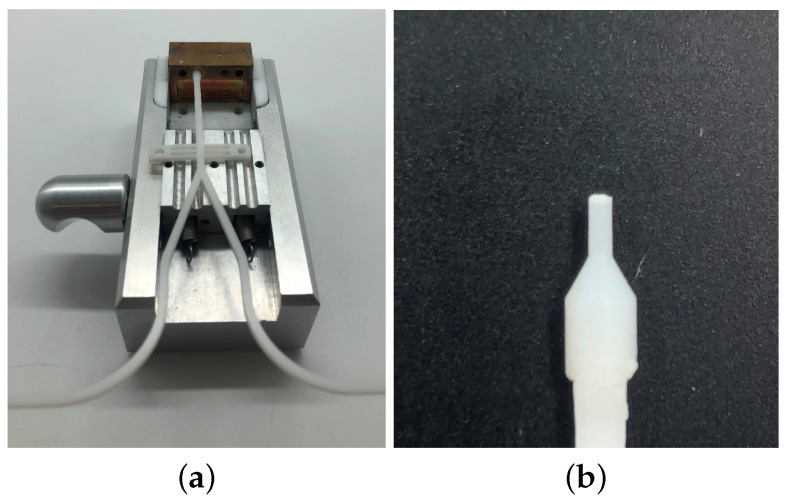
Setup for shaping the DWG end. (**a**) Mold to shape the end of the dielectric waveguide. (**b**) Dielectric waveguide with end fused in shape.

As the reflectometer, we used the ZNA from Rhode & Schwarz, centered at 82.5 GHz, with a bandwidth of 15 GHz. From the VNA, the wave is guided into the coupling via rectangular waveguides. The coupling is also made with the help of a 3D printer. To facilitate the production, the horn is divided into two parts, which are fixed with screws. The tip of the dielectric waveguide, which is placed in the cone-shaped milled coupling structure, is melted into the appropriate shape using the mold shown in Figure 25a. For this purpose, the piece of metal made of copper is heated and the waveguide is placed into the slot provided for this purpose. Slowly, the waveguide is pushed into the warm copper piece, working very precisely so that the tip is centered and straight.

The waveguides used for the measurement are printed with the 3D printer i3 MK3 MMU2 from the manufacturer Prusa with a filament made of high-impact polystyrene (HIPS). HIPS is characterized by its low loss factor.

### 6.2. Measurement Results

**Figure 26:**
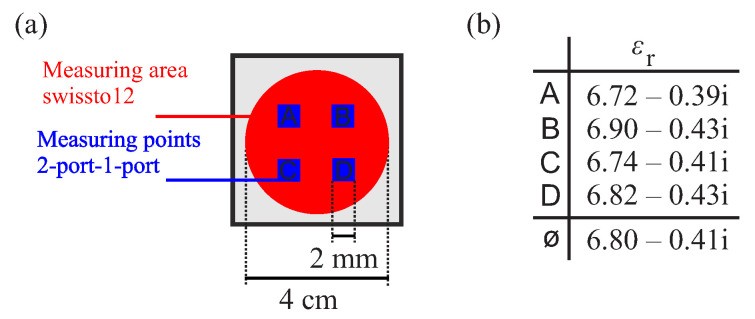
(**a**) Schematic drawing of the position and size of the four measuring points A, B, C, and D. (**b**) Permittivity at the measuring points for a MUT with an 18% ${\mathrm{BaTiO}}_{3}$ concentration.

To minimize undesirable properties and the resulting measurement uncertainties, a TLR calibration [16] is performed in advance. Subsequently, different materials with different permittivities were measured. The material samples were mixed independently and consist of a mixture of epoxy resin and barium titanate in various concentrations. Using the 2-port-1-port setup, each sample was measured at four positions. Figure 26a shows a schematic drawing of the arrangement and size of the measuring points in relation to the measuring area of the swissto12 setup. Measurements at four different positions increase the comparability with the measured values from the swissto12 setup, especially for inhomogeneous samples. To illustrate the inhomogeneity of the MUTs, the permittivity at the individual measuring points is given as an example in Figure 26b, for a barium titanate concentration of 18%. To determine the permittivity $\varepsilon_{r}$ using the NRW and Baker–Jarvis algorithms, the S-parameters resulting from the measurement were used. An average permittivity $⌀\varepsilon_{r}$ was calculated from the four measurements per sample. For comparison, the permittivity $\varepsilon_{r_{2}}$ of the same samples was measured again using a verified measurement method from swissto12. The results were averaged over the frequency and listed in Table 1.

The results for the relative dielectric constant are very similar for both measurements. With a max. error of 1.76% for the present investigation, the applicability and accuracy of the proposed measurement system is demonstrated.

The main differences in the extraction of the material parameters between the two measurement setups occur in the extraction of losses. The imaginary part has an maximum error of about 12.5%. This may be due to the fact that the ports are not exactly aligned and, on the other hand, due to scattering at the MUT.

## 7. Conclusions

In this contribution, an optimized form of the 2-port-1-port system was shown, which now has mounting and support structures. Moreover, a novel coupling was designed, which creates a low-reflection transition between the waveguide and the dielectric waveguide. Due to an optimized dielectric waveguide system, the novel coupling, and the use of a broadband VNA, it is possible to acquire the complete S-parameter set with only a one-port measurement. The algorithm adapted to the system provides the permittivity from the S-parameter. Three-dimensional EM simulations, as well as real measurements proved the applicability of the proposed system. Future work will investigate improving the accuracy of the 2-port-1-port-system.

## 8. Discussion

In this paper, we present a measurement system for material characterization in the millimeter-wave range. The system allows measuring extremely small samples with an area of $0.25\;{\mathrm{cm}}^{2}$, compared to other methods, which require $16\;{\mathrm{cm}}^{2}$. This is a factor of 64, which is particularly disadvantageous for very small and very expensive materials. Using a dielectric waveguide, it is possible to measure the complete S-parameters with only one port, so that a measurement can also be made using radar. Radar-based systems are advantageous compared to vector network analyzers (VNAs) because they can be implemented very compactly and are much less expensive than laboratory instruments. Overall, the 2-port-1-port system is significantly less expensive compared to currently available measurement systems. This is due, on the one hand, to the simple production using 3D printers and, on the other hand, to the low-cost material from which the waveguide is made. In terms of measurement accuracy, the results for the relative permittivity showed a maximum error of 1.76% compared to the measurements with the MCK from Swissto12. The main differences in the extraction of the material parameters between the measurement setups occur in the extraction of the losses. The imaginary part has an error of about 12.5%. This may be due to the fact that the connections are not precisely aligned. Due to the flexible material of the waveguide, the setup is not as stable and robust as waveguide systems. All in all, the measurement method is an inexpensive alternative, which is easy to manufacture and provides accurate measurement results with real part permittivity.

## Figures and Tables

**Table 1:** Material characterization results from the 2-port-1-port setups and the swissto12 setup.

${\mathrm{BaTiO}}_{3}$	Thickness	$⌀\varepsilon_{r}$	$\varepsilon_{r_{2}}$	$\Delta \varepsilon^{\prime }$	$\Delta \varepsilon^{\prime \prime }$
Conc.%	μm	(2-port-1-port)	(MCK Swissto12)	%	%
4	2050	3.38 − 0.11i	3.39 − 0.10i	−0.3	10
6	2060	3.91 − 0.13i	3.98 − 0.12i	−1.76	8.3
8	4920	4.44 − 0.19i	4.40 − 0.17i	0.9	11.8
10	3989	5.00 − 0.26i	5.01 − 0.23i	−0.20	8.0
12	4000	5.47 − 0.29i	5.53 − 0.26i	−1.08	11.5
14	4000	5.97 − 0.32i	5.99 − 0.29i	−0.33	10.3
15	3210	6.39 − 0.35i	6.38 − 0.33i	0.16	6.6
17	5110	6.46 − 0.36i	6.45 − 0.32i	0.15	12.5
18	5060	6.80 − 0.41i	6.80 − 0.38i	−0.00	7.9
